# Lithium: Managing Cognitive Impairment and Sexual Problems

**DOI:** 10.1192/j.eurpsy.2023.2141

**Published:** 2023-07-19

**Authors:** J. Petta, A. L. Falcão, G. Soares, A. Lourenço

**Affiliations:** 1 Centro Hospitalar Psiquiátrico de Lisboa, Lisboa, Portugal

## Abstract

**Introduction:**

Patients taking lithium complain of cognitive impairment. This was assumed to be real by expert clinicians for years until relatively recent objective neuropsychological studies have failed to verify much impairment.

Second and perhaps underemphasized side effect from lithium is sexual dysfunction.

**Objectives:**

The objective of this review is to highlight for the cognitive and sexual problems, which are two very important areas for discussion with patients. It should be brought up right when beginning to prescribe.

**Methods:**

Data was obtained through an internet-based literature review, using the research platform PubMed and the World Health Organization website. Eight articles from the last five years were included.

**Results:**

Due to the lack of evidence in neuropsychological studies, what was considered to be impaired cognitive function in the past has been recently considered a loss of sharp thinking in manic states or mild persisting depressions.

About sexual dysfunction it is important eliminating other possible causes, lowering lithium dose, timing sex, and taking sildenafil and 240 mg/day of aspirin may help.

**Conclusions:**

Cognitive impairment and sexual problems are two important subjects that involve the issues of dosing, of managing and dealing with people’s willingness to take lithium.

Providing psychoeducation about these possible effects can head off abrupt discontinuation impulses.

**Disclosure of Interest:**

None Declared

